# Effect of sidedness on survival among patients with early-stage colon cancer: a SEER-based propensity score matching analysis

**DOI:** 10.1186/s12957-021-02240-3

**Published:** 2021-04-19

**Authors:** Zhuang-Sheng Huang, Jun-Wei Wu, Ying Li, Yu-Hai Lin, Xu-Yuan Li

**Affiliations:** 1 Department of General Surgery, Shantou Central Hospital, Shantou, Guangdong China; 2 Department of Medical Oncology, Shantou Central Hospital, Shantou, Guangdong China

**Keywords:** SEER database, Survival, Sidedness, Colon cancer, Propensity score matching, Laterality

## Abstract

**Background:**

Most previous studies compared survival between left-sided and right-sided colon cancer without adjustment for clinicopathological parameters. We investigated the effect of sidedness on survival among patients with early-stage colon cancer, using a propensity score matching method.

**Methods:**

The 18 registry custom data within the SEER database were used to identify patients who were diagnosed with colon cancer between 2010 and 2014. A propensity score matching analysis was performed using the nearest neighbor method. Survival was estimated using the Kaplan–Meier method. A Cox proportional hazards model was applied to determine the prognostic factors.

**Results:**

In the unmatched cohort, 25,094 (35.72%) patients were diagnosed with left-sided colon cancer and 45,156 (64.28%) with right-sided colon cancer. After propensity score matching, each cohort included 5118 patients, and the clinicopathological characteristics were well balanced. In the unmatched cohort, left-sided colon cancer had superior all-cause (*χ*^2^=315, *P*<0.01) and cancer-specific (*χ*^2^=43, *P*<0.01) survival than right-sided tumors. However, in the matched cohort, no difference was observed for all-cause (*χ*^2^=0.7, *P*=0.4) and cancer-specific (*χ*^2^=0, *P*=0.96) survival between left and right colon cancer. The Cox model did not indicate sidedness as a prognostic factor. In the subgroup analysis, stage II right-sided colon cancer had a better survival outcome, while stage III left-sided tumors had a better survival outcome.

**Conclusions:**

After adjusting for clinicopathological characteristics in this study, sidedness showed no impact on survival in early-stage colon cancer. However, sidedness was associated with prognostic differences in stages II and III early-stage colon cancer.

**Supplementary Information:**

The online version contains supplementary material available at 10.1186/s12957-021-02240-3.

## Background

The primary tumor location in metastatic colon cancer has gradually become a significant factor to be integrated into the treatment plan, with evidence from post hoc analyses of clinical trials and meta-analysis indicating that sidedness in metastatic colon cancer is a strong prognostic parameter [1–3]. Right-sided metastatic colon cancer is associated with a poorer survival rate than left-sided metastatic colon cancer [4, 5].

Several studies have investigated the impact of sidedness on survival among patients with early-stage colon cancer but have reported inconsistent results [6–9]. Some studies showed no difference in survival between left- and right-sided diseases [6, 7], while others demonstrated a survival difference in operable colon cancer [8, 9]. However, the prognostic value of sidedness in studies which reported a difference is conflicting, and a subgroup analysis based on stage showed a prognostic difference. Above all, nearly all studies based on the Surveillance, Epidemiology and End Results (SEER) database did not achieve a balance of clinicopathological characteristics before performing survival analyses between left- and right-sided colon cancer.

In this study, we aimed to explore the effect of sidedness on survival among patients with stage I–III colon cancer, using a propensity score matching method to balance baseline characteristics including age, race, sex, tumor grade, T stage, N stage, and TNM stage, based on the SEER database.

## Methods

This was a retrospective, observational study. The SEER database collects data on patients diagnosed with cancers, using population-based registries, which represent approximately 28% of the population of the USA. Data collected include patient demographics, tumor sites, histologic information, treatments (surgery, radiotherapy, and chemotherapy), staging, follow-up, vital status, and survival time.

The 18 registry custom data (with additional treatment fields) within the SEER database were used in this study to identify patients who were diagnosed with colon cancer between 2010 and 2014.

The inclusion criteria were as follows: patients aged 18 years or older; patients whose tumor had a malignant behavior and had radical surgery; patients with histology codes (International Classification of Diseases for Oncology, 3rd Edition, ICD-0-3) of 8140, 8141, 8143, 8144, 8147, 8210, 8211, 8246, 8255, 8260, 8261, 8622, 8263, 8480, 8481, 8490, 8510, 8560, and 8574; patients with site codes of C18.0 (cecum), C18.2 (ascending colon), C18.3 (hepatic flexure of the colon), C18.4 (transverse colon), C18.5 (splenic flexure of the colon), C18.6 (descending colon), and C18.7 (sigmoid colon); patients who were under active follow-up; and patients for whom complete dates were available.

The exclusion criteria were as follows: rectal cancer or unknown primary site; M1 disease; stage 0, stage IV, in situ tumor, or unknown tumor stage; younger than 18 years; incomplete dates of follow-up; inadequate information for staging (T*x*, N*x*); and unknown tumor grade (G*x*).

Right-sided colon cancer included cancers originating in the cecum, ascending colon, hepatic flexure, and transverse colon. Left-sided colon cancer included cancers located primarily in the splenic flexure, descending colon, and sigmoid colon. Patients were subdivided into three groups according to age: <50, 50–69, and ≥70 years. Tumor staging was performed according to the guidelines of the American Joint Committee on Cancer (AJCC) 6th edition.

For propensity score matching, we identified 5118 patients diagnosed with left-sided colon cancer in 2010 and sought a matched cohort from 45,156 patients with right-sided tumors who were diagnosed from 2010 to 2014, using the one-to-one nearest neighbor method. Matching was performed using clinicopathological factors including age, race, sex, tumor grade, T stage, N stage, and TNM stage.

All-cause survival and cancer-specific survival rates were estimated using the Kaplan–Meier method and compared using the log-rank test. Categorical data were analyzed using the chi-squared test, and continuous data were analyzed using the Student *t* test. Prognostic factors were determined using the Cox proportional hazards model. All statistical analyses were performed using R version 3.3.2 (R Foundation for Statistical Computing, Vienna, Austria). Analysis items with two-sided *P* < 0.05 were considered statistically significant.

## Results

After screening, a total of 70,250 patients were included in the final analysis. Among them, 25,094 (35.72%) patients were diagnosed with left-sided colon cancer and 45,156 (64.28%) with right-sided colon cancer. The AJCC stage distributions for stages I, II, and III were 26.83%, 36.83%, and 36.34%, respectively. Before propensity score matching, there was an imbalance across all the clinicopathological characteristics. Right-sided colon cancer relatively tended to occur in patients older than 70 years (57.66% vs. 38.82%), in the white population (81.28% vs. 76.90%), and in patients whose tumors were poorly differentiated (grades III–IV, 22.51% vs. 13.14%). After propensity score matching, each cohort included 5118 patients, and the clinicopathological characteristics were well balanced (Table 1). Neither the median all-cause survival nor the median cancer-specific survival was reached for left- or right-sided colon cancer in the entire and the matched cohorts.

**Table 1:** Characteristics of included patients

	Before matching, *n* (%)		*P*	After matching, *n* (%)		*P*
	Left colon, *n*=25,094	Right colon, *n*=45,156		Left colon, *n*=5118	Right colon, *n*=5118	
Age, years (median)	65	72	<0.01	66	66	0.91
Age groups			<0.01			0.86
<50	3057 (12.18)	2770 (6.13)		626 (12.23)	608 (11.88)	
50–69	12,296 (49.00)	16,350 (36.21)		2428 (47.44)	2438 (47.64)	
≥70	9741 (38.82)	26,036 (57.66)		2064 (40.33)	2072 (40.48)	
Sex			<0.01			0.74
Male	13,451 (53.60)	20,708 (45.86)		2720 (53.15)	2738 (53.50)	
Female	11,634 (46.36)	24,448 (54.14)		2398 (46.85)	2380 (46.50)	
Race			<0.01			0.29
White	19,297 (76.90)	36,705 (81.28)		3995 (78.06)	4041 (78.96)	
Black	2934 (11.69)	5382 (11.92)		591 (11.55)	586 (11.45)	
Others	2756 (10.98)	2935 (6.50)		518 (10.12)	484 (9.45)	
Unknown	107 (0.43)	134 (0.30)		14 (0.27)	7 (0.14)	
Grade			<0.01			0.66
I	2329 (9.28)	3858 (8.54)		506 (9.89)	525 (10.26)	
II	19,469 (77.58)	31,131 (68.94)		3882 (75.85)	3892 (76.05)	
III	2802 (11.17)	8288 (18.35)		643 (12.56)	627 (12.25)	
IV	494 (1.97)	1879 (4.16)		87 (1.70)	74 (1.44)	
T			<0.01			0.73
T1	4402 (17.54)	5879 (13.02)		912 (17.82)	895 (17.49)	
T2	4019 (16.02)	7938 (17.58)		843 (16.47)	855 (16.71)	
T3	13,407 (53.43)	25,277 (55.98)		2765 (54.03)	2801 (54.73)	
T4	3266 (13.02)	6062 (13.42)		598 (11.68)	567 (11.07)	
N			<0.01			0.54
N0	15,479 (61.68)	29,242 (64.76)		3184 (62.21)	3226 (63.03)	
N1	6684 (26.64)	10,350 (22.92)		1336 (26.10)	1327 (25.93)	
N2	2931 (11.68)	5564 (12.32)		598 (11.69)	565 (11.04)	
Stage			<0.01			0.85
I	6938 (27.65)	11,912 (26.38)		1458 (28.49)	1469 (28.70)	
II	8541 (34.04)	17,330 (38.38)		1726 (33.72)	1757 (34.33)	
III	9615 (38.32)	15,914 (35.24)		1934 (37.79)	1892 (36.97)	

### All-cause survival

**Fig. 1:**
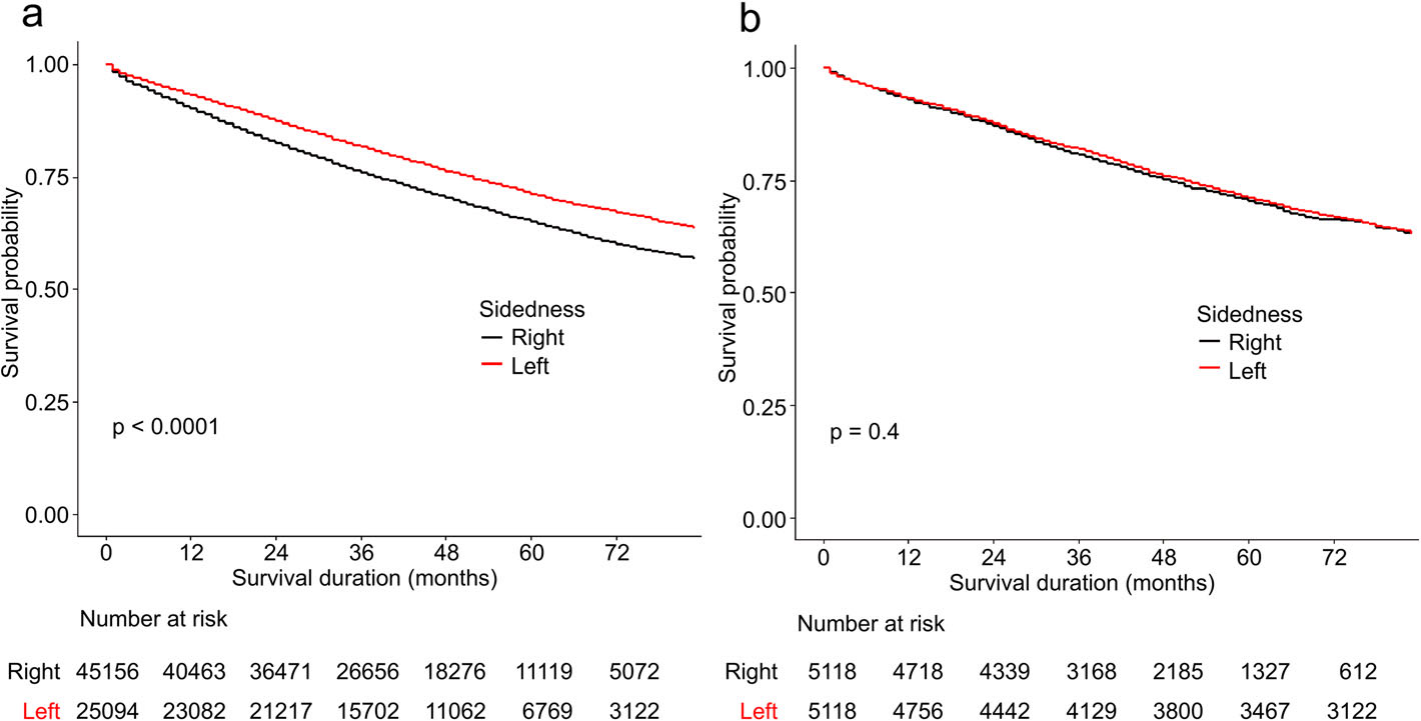
All-cause survival curves for the unmatched (**a**) and matched cohorts (**b**)

**Fig. 2:**
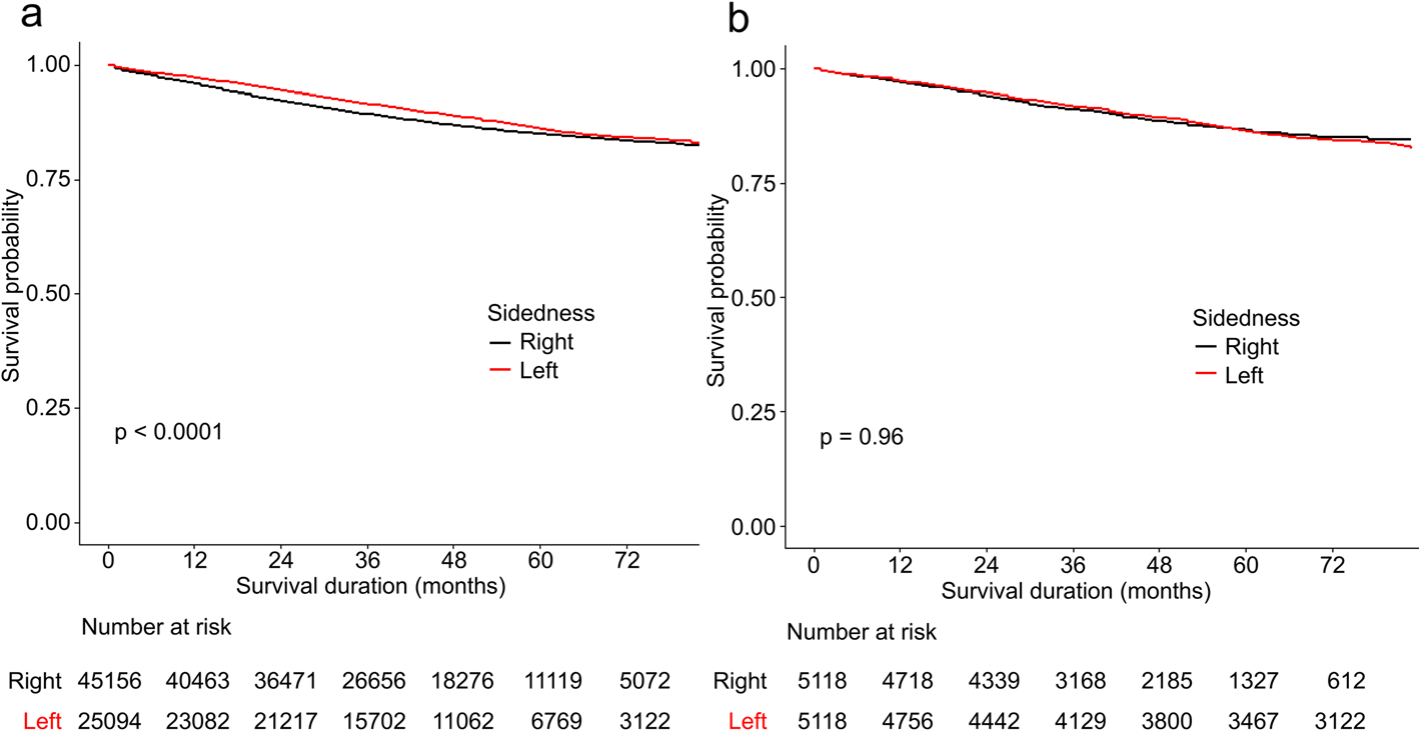
Cancer-specific survival curves for the unmatched (**a**) and matched cohorts (**b**)

For all-cause survival in the entire cohort, patients with left-sided tumors had superior outcomes than did those with right-sided tumors (*χ*^2^=315, *P*<0.01) (Fig. 1a), and the superiority was observed across all stages (I to III) (Fig. 2a). The univariate Cox proportional hazards model showed a reduction in mortality risk with left-sided colon cancer (HR=1.31, 95% confidence interval 1.27–1.35, *P*<0.01). However, sidedness was not found to be a prognostic factor in the multivariate hazards model (HR=0.98, 95% confidence interval 0.95–1.00, *P*=0.08) (Table 2).

**Table 2:** Cox proportional hazards analysis of all-cause survival

	Unmatched cohort			*P*	Matched cohort			*P*
	HR	95% CI			HR	95% CI		
Univariate								
Age	1.047	1.046	1.049	<0.001	1.047	1.044	1.050	<0.001
Age groups	2.221	2.164	2.280	<0.001	2.267	2.131	2.411	<0.001
Race	0.892	0.871	0.913	<0.001	0.940	0.889	0.994	0.031
Sex	0.972	0.945	0.999	0.04	0.860	0.800	0.923	<0.001
Grade	1.423	1.392	1.453	<0.001	1.351	1.268	1.439	<0.001
T	1.597	1.569	1.626	<0.001	1.565	1.497	1.636	<0.001
N	1.568	1.540	1.596	<0.001	1.520	1.451	1.593	<0.001
Stage	1.533	1.505	1.562	<0.001	1.509	1.441	1.580	<0.001
Sidedness	1.311	1.272	1.350	<0.001	0.969	0.901	1.043	0.4
Multivariate								
Age	1.049	1.048	1.050	<0.001	1.049	0.954	1.046	<0.001
Race	0.972	0.950	0.996	0.0198	0.968	0.916	1.023	0.253
Sex	0.836	0.813	0.860	<0.001	0.824	0.767	0.885	<0.001
Grade	1.230	1.203	1.258	<0.001	1.261	1.180	1.348	<0.001
Stage	1.584	1.554	1.615	<0.001	1.550	1.477	1.626	<0.001
Sidedness	0.977	0.947	1.007	0.0819	0.968	0.899	1.041	0.378

*HR* hazard ratio, *CI* confidence interval

In the matched cohort, no difference was observed between left and right colon cancer (*χ*^2^=0.7, *P*=0.4) (Fig. 1b). The results of the subgroup analysis stratified by stage are shown in Fig. 2b. An association between prognosis and sidedness was only observed among patients with stage III left-sided disease (*P*=0.016). In addition, both the univariate and multivariate hazards models indicated that sidedness was not a prognosticator (Table 2).

### Cancer-specific survival

**Fig. 3:**
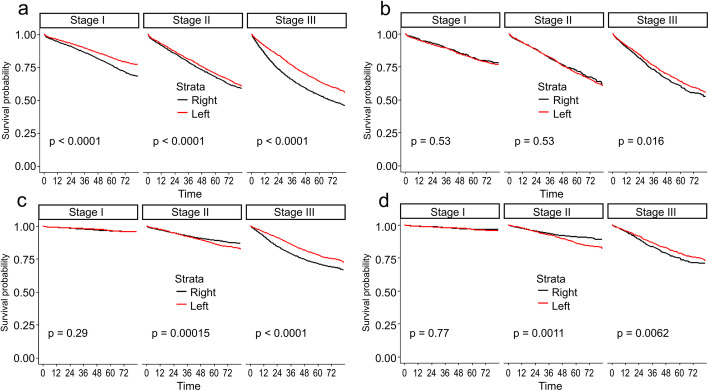
Survival curves for subgroup analysis by stage. All-cause survival curves for the unmatched (**a**) and matched (**b**) cohorts and cancer-specific survival curves for the unmatched (**c**) and the matched (**d**) cohorts

In the entire cohort, for cancer-specific survival, left colon cancer continued to demonstrate better outcomes relative to right colon cancer (*χ*^2^=43, *P*<0.01) (Fig. 3a), and the univariate hazards model supported left sidedness as a favorable factor (Table 3). However, the multivariate hazards model failed again to establish sidedness as a prognostic factor. In the stage strata, stage II right-sided colon cancer had better cancer-specific survival than did that of the left side (Fig. 3c).

**Table 3:** Cox proportional hazards analysis of cancer-specific survival

	Unmatched cohort			*P*	Matched cohort			*P*
	HR	95% CI			HR	95% CI		
Univariate								
Age	1.019	1.017	1.021	<0.001	1.017	1.012	1.021	<0.001
Age groups	1.361	1.312	1.411	<0.001	1.329	1.219	1.449	<0.001
Race	1.024	0.990	1.060	0.168	1.035	0.954	1.124	0.406
Sex	1.065	1.019	1.113	0.004	0.991	0.887	1.107	0.874
Grade	1.739	1.684	1.795	<0.001	1.701	1.552	1.866	<0.001
T	2.705	2.618	2.795	<0.001	2.438	2.252	2.640	<0.001
N	2.414	2.351	2.478	<0.001	2.325	2.172	2.488	<0.001
Stage	2.735	2.642	2.832	<0.001	2.520	2.316	2.742	<0.001
Sidedness	0.857	0.818	0.897	<0.001	0.997	0.890	1.117	1
Multivariate								
Age	1.023	1.021	1.025	<0.001	1.021	1.017	1.025	<0.001
Race	1.039	1.004	1.076	0.002	1.016	0.936	1.102	0.713
Sex	0.973	0.931	1.102	0.217	0.956	0.856	1.068	0.430
Grade	1.410	1.363	1.458	<0.001	1.444	1.310	1.529	<0.001
Stage	2.725	2.629	2.824	<0.001	2.502	2.294	2.728	<0.001
Sidedness	0.969	0.924	1.017	0.199	0.980	0.874	1.098	0.723

*HR* hazard ratio, *CI* confidence interval

In the matched cohort, the log-rank test showed no differences between left and right colon cancers (*χ*^2^=0, *P*=0.96) (Fig. 3b). In the subgroup analysis by stage, stage II right-sided colon cancer had a better survival outcome, while stage III left-sided tumors yielded improved survival (Fig. 3d). The univariate and multivariate hazards models revealed that sidedness did not affect cancer-specific survival in the matched cohort (Table 3).

## Discussion

In the present study, we captured cases from the SEER database and compared survival between right-sided and left-sided colon cancer, before and after propensity score matching. For the entire cohort, our findings were in line with previous reports [8, 9], showing that left-sided colon cancer demonstrated superior all-cause and cancer-specific survival. The superiority of survival (all-cause and cancer-specific) for left-sided colon cancer, however, was not sustained in the matched cohort. In addition, the Cox model did not validate the prognostic value of sidedness in either cohort (unmatched or matched). No survival difference was observed between left- and right-sided colon cancer in the matched cohort in our study. However, subset analysis according to stage showed that stage III left colon cancer was associated with improved all-cause and cancer-specific survival, which was consistent with the findings of previous studies [8–10]. For stage II disease in the matched cohort, patients with right-sided tumor had superior cancer-specific survival, which was also in keeping with the findings of other studies [8–10].

Some known clinicopathological parameters, such as age, tumor grade, and stage, are strongly related to survival in colon cancer. In most previous SEER-based studies, baseline characteristics of left- and right-sided colon cancers were not balanced. Therefore, we deemed the adjustment of these baseline parameters among the right- and left-sided tumors as a prerequisite before a survival comparison. Along with our study, one study conducted in 2016 applied the propensity score matching method to balance the baseline characteristics [10], but the stage distribution in this study, probably the most prognostic factor, was unknown. For the matched cohort in our study, all the included parameters were comparable between the two groups.

Some studies reported contradictory results regarding the prognostic value of sidedness in colon cancer. In a Canadian population-based cohort study that involved over 6000 patients, the location of the primary tumor was not associated with overall survival or cancer-specific survival in early-stage colon cancer. In addition, laterality was not associated with prognosis in patients with stage III disease [6]. An important strength of the Canadian study was the adjustment for comorbidity, as right-sided colon cancer was more likely to be diagnosed at an advanced age. It should be noted that all SEER-based analyses, including ours, were not able to adjust for comorbidity.

In studies that used the SEER database, the results of stage-matched comparisons among right- and left-sided colon cancer were similar to ours: right-sided tumors showed better survival in stage II disease but showed worse survival in stage III disease. The underpinnings of this inconsistency were not fully understood. Lateralized colon cancer was known to demonstrate high microsatellite instability (MSI) [11, 12], and colon cancers with high MSI had significantly better prognoses [13, 14]. The frequency of MSI positivity was significantly higher with stage II disease than with stage III disease [15]. The status of MSI might partly explain this observation.

Molecular subgroups of colon cancers exhibit discrepancy in survival. *BRAF* mutation, SMAD4 loss, and *KRAS* mutation are all associated with inferior survival among patients with colon cancer [16–18]. However, similar to all other SEER-based studies, our study was unable to determine molecular subgroups.

Our study had some limitations. Firstly, we were unable to include information on other factors that are known to affect survival in colon cancer, such as performance status, use of adjuvant chemotherapy, and comorbidity, due to the retrospective nature of the study. Unbalance of these factors between the left and right early-stage colon cancer could still be an important bias for this study. Secondly, molecular-level information was not available as well, which could be the inherent mechanism for survival discrepancy. Using a large real-world database with molecular information to further explore the impact of sidedness seems to be a feasible way, and further investigation should be made. Finally, it was unknown whether our results shed light on clinical practice, e.g., for stage II colon cancer, the primary location of tumor currently is not an evident factor in decision-making for adjuvant treatment. Given these limitations, this study did not have new data from the SEER database to present new findings.

However, the strength of our study was the use of the propensity score matching method to balance some important prognostic factors such as stage, grade, and age. Our results from the matched cohort strengthened the findings of other SEER-based analyses that the sidedness indeed played different roles in stages II and III colon cancer, which might inform the trial design in adjuvant chemotherapy. Recent evidence suggested that for stage III disease, 3 months of adjuvant chemotherapy might be enough for patients at low risk of recurrence (T1, T2, or T3 and N1). However, future trials focusing on adjuvant chemotherapy should stratify such low-risk patients by laterality.

In conclusion, after adjusting for the clinicopathological characteristics included in our study, sidedness showed no impact on survival in early-stage colon cancer. However, sidedness was associated with prognostic differences in stages II and III early-stage colon cancer.

## Supplementary Information

**Additional file 1.**

**Additional file 2.**

## Data Availability

The datasets used and analyzed during the current study are available from the corresponding author on reasonable request.
