# Supplementary material for: Effect of sidedness on survival among patients with early-stage colon cancer: a SEER-based propensity score matching analysis
Source: World J Surg Oncol. 2021 Apr 19;19:127. doi: 10.1186/s12957-021-02240-3 (PMC8056525; doi:10.1186/s12957-021-02240-3)
Supplement: Supplementary file 1 — Additional file 1. [file 12957_2021_2240_MOESM1_ESM.docx]

Flow diagram of the Study

Potential Cases:

1. Patients whose tumor had a malignant behavior and had radical surgery.

2. With histology codes

3. Under active follow-up

Cases excluded:

1. Rectal cancer or unknown primary site

2. Younger than 18 years

3. Unknown tumor stage

4. Incomplete dates of follow-up

5. Unknown tumor grade (Gx)

6. Specific cause of death not available, not first tumor

7. M1 disease

Histologic diagnosis of resected colon Cancers 2010-2014

N =70,250

Right sided colon cancer

N =45,156

Left sided colon cancer

N =25,094
