# Supplementary material for: Effect of sidedness on survival among patients with early-stage colon cancer: a SEER-based propensity score matching analysis
Source: World J Surg Oncol. 2021 Apr 19;19:127. doi: 10.1186/s12957-021-02240-3 (PMC8056525; doi:10.1186/s12957-021-02240-3)
Supplement: Supplementary file 2 — Additional file 2. [file 12957_2021_2240_MOESM2_ESM.docx]

**Author Information**

| **Order of Author (s)** | **First**  **Name** | **Last**  **Name** | **Academic**  **Degree(s)** | **E-mail**  **Address(s)** | **Phone number** |
| --- | --- | --- | --- | --- | --- |
|  | Zhuang-Sheng | Huang | M.D | 674032684@qq.com | 8613750432845 |
|  | Jun-Wei | Wu | M.D | wujunweist@163.com | 8613632255389 |
|  | Ying | Li | M.D | 123965215@qq.com | 8613818414567 |
|  | Yu-Hai | Lin | M.D | 1379998007@qq.com | 8613600113999 |
|  | Xu-Yuan | Li | M.D | lxuyuan@qq.com | 8613592882093 |
